# Pleiotropic Control by Testosterone of a Learned Vocal Behavior and Its Underlying Neuroplasticity^1^,^2^,^3^

**DOI:** 10.1523/ENEURO.0145-15.2016

**Published:** 2016-01-23

**Authors:** Beau A. Alward, Farrah N. Madison, Shannon E. Parker, Jacques Balthazart, Gregory F. Ball

**Affiliations:** 1 Department of Psychological and Brain Sciences, The Johns Hopkins University, Baltimore, Maryland 21218; 2 Department of Psychology, University of Maryland, College Park, Maryland 20742; 3 GIGA Neuroscience, University of Liège, 4000 Liège, Belgium

**Keywords:** social behavior, songbirds, neurogenesis, singing behavior, medial preoptic area

## Abstract

Steroid hormones coordinate multiple aspects of behavior and physiology. The same hormone often regulates different aspects of a single behavior and its underlying neuroplasticity. This pleiotropic regulation of behavior and physiology is not well understood. Here, we investigated the orchestration by testosterone (T) of birdsong and its neural substrate, the song control system. Male canaries were castrated and received stereotaxic implants filled with T in select brain areas. Implanting T solely in the medial preoptic nucleus (POM) increased the motivation to sing, but did not enhance aspects of song quality such as acoustic structure and stereotypy. In birds implanted with T solely in HVC (proper name), a key sensorimotor region of the song control system, little or no song was observed, similar to castrates that received no T implants of any sort. However, implanting T in HVC and POM simultaneously rescued all measures of song quality. Song amplitude, though, was still lower than what was observed in birds receiving peripheral T treatment. T in POM enhanced HVC volume bilaterally, likely due to activity-dependent changes resulting from an enhanced song rate. T directly in HVC, without increasing song rate, enhanced HVC volume on the ipsilateral side only. T in HVC enhanced the incorporation and recruitment of new neurons into this nucleus, while singing activity can independently influence the incorporation of new neurons into HVC. These results have broad implications for how steroid hormones integrate across different brain regions to coordinate complex social behaviors.

## Significance Statement

Successful performance of social behaviors requires the coordination of multiple cognitive and physiological features. While steroid hormones are involved in the coordination of these features, it is unclear how. This study shows in canaries (*Serinus canaria*) that testosterone (T) in the medial preoptic nucleus regulates the motivation to sing, while T in the premotor song nucleus HVC regulates the quality of song. T in HVC enhanced the recruitment of new neurons into this nucleus, while singing activity may also influence the incorporation of new neurons into HVC. These results have broad implications for how steroid hormones integrate across different brain regions and via different cellular events to coordinate complex social behaviors, especially those dependent on experience.

## Introduction

Steroid hormones such as testosterone (T) regulate multiple aspects of physiology, morphology, and behavior (McEwen et al., 1991; Ball et al., 2002; Lee and Pfaff, 2008; Adkins-Regan, 2009; Brenowitz, 2015). This pleiotropic regulation by steroids coordinates suites of traits into adaptive responses to a need or challenge (Arnold, 1981; Lee and Pfaff, 2008; Pfaff et al., 2008). Different aspects of a specific behavior, including motivation, performance, cognition, and the underlying neural circuits, are often controlled by the same hormone (Alexander and Sherwin, 1991; Brenowitz and Lent, 2002; Meitzen et al., 2007; Pfaff et al., 2008; Alward et al., 2013; Kelly et al., 2014), but it is unclear how steroid hormones orchestrate such an integration. Here, we investigated the regulation by T of birdsong and its neural substrate to illuminate how such a multifactorial regulation can ensue.

A network of interconnected brain nuclei, the song control system (SCS), orchestrates song learning and production (Nottebohm et al., 1976; Vu and Mazurek, 1994; Fee and Scharff, 2010; Brainard and Doupe, 2013). HVC (proper name) and the robust nucleus of the arcopallium (RA) regulate the production of song, while Area X and the lateral magnocellular nucleus of the anterior nidopallium (LMAN) are involved in the auditory feedback used to learn and maintain adult song. RA projects to hindbrain nuclei that control respiration and the syrinx, the avian vocal organ, to generate song. These forebrain nuclei undergo remarkable plasticity in response to seasonally changing concentrations of T (Nottebohm, 1981; Bernard and Ball, 1995; Tramontin and Brenowitz, 2000; Brenowitz and Lent, 2002; Thompson et al., 2007; Hurley et al., 2008; Meitzen and Thompson, 2008). Other factors, including singing activity, social cues, and photoperiod, can contribute to the occurrence of seasonal neuroplasticity independently of T (for review, see Ball et al., 2002, 2004). The distinct roles of these nuclei in the regulation of song behavior and their robust neuroplasticity in correlation with social needs have made songbirds an excellent model in which to study the neural bases of complex learned motor behaviors as well as the regulation of their substrate (Ball et al., 2002; Fee and Scharff, 2010).

Substantial seasonal variation in the features of birdsong has been observed (Catchpole and Slater, 2003; Meitzen and Thompson, 2008). During the nonbreeding season, when T is low, songbirds such as canaries (*Serinus canaria*) and white-crowned sparrows (*Zonotrichia leucophrys*) sing with less acoustic stereotypy (i.e., less consistency from rendition to rendition) compared with the breeding season, when T is high (Nottebohm et al., 1986; Smith et al., 1997). While in a breeding state, unpaired male canaries enhance the complexity and loudness of song (Alward et al., 2014). These changes in song quality are presumably driven by the actions of T (Gil and Gahr, 2002; Sakata and Vehrencamp, 2012). T treatment of castrated male canaries enhances stereotypy, complexity, and loudness of song (Alward et al., 2013; Madison et al., 2015), all of which enhance the effectiveness of song in attracting a mate (Botero et al., 2009; Byers et al., 2010; Sakata and Vehrencamp, 2012).

Androgen receptors are expressed in HVC, RA, LMAN, and throughout the hypothalamus and midbrain, while estrogen receptors are expressed in HVC of some species as well as in the hypothalamus (Bernard et al., 1999; Metzdorf et al., 1999; Ball et al., 2002). The presence of androgen receptors in the SCS, together with evidence correlating T secretion to song output, suggested that T acts directly in the SCS to activate song behavior (Harding et al., 1988; DeVoogd 1991; Ball et al., 2002). However, recent evidence suggests that the role of T in the activation of song is not so simple. For instance, T action in the medial preoptic nucleus (POM) promotes singing motivation (song rate) but not song energy (loudness or amplitude) or quality (Alward et al., 2013). Furthermore, androgenic or estrogenic action in HVC does not enhance the song rate of castrated white-crowned sparrows (Brenowitz and Lent, 2002), but androgen or estrogen receptor blockade in HVC reduces song stereotypy (Meitzen et al., 2007).

We show here that castrated male canaries implanted with T solely in POM display enhanced motivation to sing, but not enhanced song complexity and stereotypy; however, implanting T in the HVC of these same birds rescued all of these song measures. T acting directly in HVC enhanced the recruitment of new neurons into this nucleus, while singing activity also seems to influence the incorporation of new neurons into HVC. These results illustrate how steroid hormones can have different consequences in different brain areas to organize complex social behaviors and regulate their neural substrate.

## Materials and Methods

### Animals and pre-experimental manipulations

Canaries (*S. canaria*) of the Border strain were used for this study. Male and female canaries were obtained from a local breeder (Maryland Exotic Birds). Upon entry into the laboratory, birds were placed on a short day photoperiod (8 h light/16 h dark) for 6 weeks to induce a state of photosensitivity (Nicholls and Storey, 1977; Hurley et al., 2008). Birds in a photosensitive but not photostimulated state also possess regressed testes, which facilitates effective castration. All procedures were approved by The Johns Hopkins University Animal Care and Use Committee.

For castration, male birds were deeply anesthetized with isoﬂurane gas (IsoSol isoﬂurane, Vedco; Isotec 4 anesthesia machine, SurgiVet) and placed on their right side. The left testis was then removed through an incision below the last rib; immediately after, the bird was placed on its left side, and the right testis was removed in an identical manner. After recovery from surgery, birds were placed under a heat lamp until they perched. Then, birds were placed back in their home cage and allowed to recover for 6 weeks, to allow adequate time for the physiological and behavioral effects of T to disappear.

### Experimental groups and stereotaxic implantation

All birds were implanted with two brain cannulae targeting HVC and POM ipsilaterally, and a T-filled or empty Silastic subcutaneous implant; the contents of cannulae or implants varied based on the treatment group. Birds were anesthetized using isoflurane gas and immediately implanted under the skin above the wing shoulder with a Silastic implant (10 mm in length; Dow Corning; outside diameter, 1.65 mm; inside diameter, 0.76 mm). Birds were then placed in a stereotaxic apparatus modified for use in small birds such as canaries with the beak holder placed 45° below the horizontal axis of the apparatus. We used the following stereotaxic coordinates to target POM: dorsoventral, −7 mm from the dorsal surface of the brain; anteroposterior, 2.3 mm from the rostral tip of the cerebellum; and mediolateral, ±0.15 mm from the midline. For HVC, the following coordinates were used: dorsoventral, −0.60 mm from the dorsal surface of the brain; anteroposterior, −0.20 mm from the rostral tip of the cerebellum; and mediolateral, ±2.70 mm from the midline. Each bird received ipsilateral, unilateral implants aimed at POM and HVC using a Hamilton syringe fashioned to hold the 27 gauge cannula filled with T or left empty. Cannulae were lowered to the target coordinates, and dental cement was applied around the implant. The excess portion of the cannula was clipped off after the cement had dried. The skin was then sutured over the implant, and lidocaine and antibiotics were applied around the sutured portion of the skin using a Q-tip.

Implants were made using blunted 27 gauge needles filled over a length of 1 mm with crystalline T (Balthazart and Surlemont, 1990; Alward et al., 2013; Sigma T 1500) or left empty as a control. Implants were cleaned using acetone and a Kimwipe to remove any hormone that adhered to the outside of the tube. The side of the brain in which the implants were placed was randomized across birds. Once birds recovered, they were returned to individual sound-attenuated chambers set to a photoperiod of 14 h light/10 h dark to simulate breeding photoperiods.

Due to expected variation in implant sites and the localized effectiveness of these T implants (Balthazart and Surlemont, 1990; Alward et al., 2013), we implanted 14 birds with T-filled cannulae targeting both HVC and POM, 7 birds with T targeting their POM only, 2 birds with T targeting only their HVC, and 2 birds with empty cannulae targeting both HVC and POM. Eight birds that were given T-filled subcutaneous Silastic implants were implanted with empty cannulae targeting HVC and POM. Our previous work has shown that when T missed POM, no song or copulatory behavior was induced, and the accuracy of the implants was ∼60% (Alward et al., 2013). Therefore, we lumped birds with T-filled cannulae that missed POM and birds that received empty cannulae targeting their POM into the same group, labeled the POM-NO T group, reflecting that neither group received T contacting POM. We proceeded similarly when T missed HVC in the HVC-POM T or HVC-T groups (Brenowitz and Lent, 2002; Meitzen et al., 2007). We confirmed, using unpaired *t* tests, that these subgroups that were pooled did not differ significantly on any morphological (see Materials and Methods; nuclei volumes, contralateral POM volumes), behavioral, or physiological measures [all comparisons, *p* ≥ 0.30; except for comparisons between birds with empty cannulae targeting the HVC and POM, and birds with T-filled cannulae that missed for the number of doublecortin (DCX)-immunoreactive (IR) fusiform cells in HVC and control regions (*p* ≥ 0.19)].

After histological verification of the implant locations, this experiment ended up having five groups, which were defined as follows: eight birds received T implants contacting HVC and POM (one canary in this group had testicular remnants and was excluded from the analysis; see below; HVC-POM T group, *n* = 7); eight birds received T implants contacting their POM but not their HVC (POM-T group, *n* = 8); five birds had T contacting their HVC and not their POM (HVC-T group, *n* = 5); and four birds did not have T contacting either their HVC or POM (HVC-POM NO T, *n* = 4). Birds in the HVC-T and HVC-POM NO T groups did not sing, except for one bird from each group that sang very infrequently and were thus not included in the song acoustic analysis. Eight birds received subcutaneous T implants, but one of them had testicular remnants and was removed from the analysis (see below; PER-T group, *n* = 7).

### Song recordings and features of interest

Following stereotaxic surgery, birds were placed individually in sound-attenuating recording chambers (41 × 48 × 51 cm). Isolation chambers were outfitted with a microphone (BT-MP8087 Mini Microphone, B&H Foto & Electronics Corp.) and camera (KPC-600 Pinhole Camera 3.6 mm; B&H Foto & Electronics Corp.) connected to a computer running DVRserver (version 6.33b; Mammoth Technologies) designed for real-time video and audio surveillance recording. Each day, the DVRserver captured song behavior from 8:00 to 9:30 A.M. (lights on at 8:00 A.M.), 1:00 to 2:30 P.M., and 4:00 to 5:30 P.M. in .wav files sampled at 22,050 Hz, which translated to a frequency range of 0–11 kHz. Song files were run through a high-pass filter set to a threshold of 900 Hz to remove low-frequency noise and were converted to a digital format using Goldwave (version 5.55, GoldWave) before they were visualized into sound spectrograms using Avisoft (SASlab Pro), a Windows application for investigating animal acoustic communication by increasing the efficiency in extensive sound analysis projects. For the spectrograms, the fast Fourier transform length was set to 512 with an overlap of 75% for the temporal resolution. Songs were defined as vocalizations having a duration of >1 s of continuous vocalizations with gaps no longer than 500 ms (Güttinger, 1985; Voigt and Leitner, 2008; Alward et al., 2013, 2014). Each song was verified by looking at the original sonograms to further eliminate noise that escaped the filter. Based on previous work, we used Avisoft to quantify the following song features: song rate (average number of songs/minute), energy (a measure of amplitude), maximum frequency, minimum frequency, frequency bandwidth, entropy variance, maximum frequency variance, minimum frequency variance, and bandwidth variance collapsed across the final 3 d of treatment (Alward et al., 2013; Madison et al., 2015; Rouse and Ball, 2015). Entropy is a measure of spectral width and uniformity; it is a unitless measure of signal noise; 0 would be a pure tone (Tchernichovski et al., 2000). The variance of this measure has been used in studies in zebra finches and canaries as a measure of within-song spectral diversity (Tchernichovski et al., 2000; Alward et al., 2013, 2014; Madison et al., 2015). Entropy variance and other measures of acoustic variance were validated in our laboratory as indicators of complexity in canary song by selecting 51 canary songs and analyzing them with Avisoft. In these songs, the number of different syllables was determined in each song as a measure of within-song complexity. Canaries include a variety of highly identifiable, different, discrete vocalizations within a particular song that are referred to as different syllables (Nottebohm et al., 1986). Minimum frequency variance, maximum frequency variance, and bandwidth variance correlated strongly with the number of different syllables per song (*r*_(49)_ = 0.80, *p* < 0.0001; *r*_(49)_ = 0.83, *p* < 0.0001; *r*_(49)_ = 0.86, *p* < 0.0001, respectively). Entropy variance also correlated significantly with the number of different syllables per song, but this was less strong than the other measures of complexity (*r*_(49)_ = 0.45, *p* = 0.001). Minimum and maximum frequency variance correlated strongly (*r*_(49)_ = 0.87, *p* < 0.0001), so we only report maximum frequency variance for brevity. We also include entropy variance as a measure of within-song spectral diversity given its use in previous studies.

Given the importance of producing loud signals during social–sexual interactions (Cynx and Gell, 2004; Brumm and Slater, 2006), and the function of T in regulating this acoustic feature (Alward et al., 2013; Madison et al., 2015; Murphy et al., 2015; Rouse and Ball, 2015), we quantified the energy of song, which is the integral sum of the squared amplitude of a song multiplied by its sampling time (Avisoft SASlabUser Manual).

We also quantified song stereotypy. Stereotypy has been measured in previous studies to quantify how certain acoustic features of song remain constant across multiple song renditions of a given bird (Alward et al., 2013; Meitzen et al., 2007, 2009). As mentioned above, song stereotypy is affected by the actions of steroid hormones, and females prefer higher song stereotypy (Alward et al., 2013; Meitzen et al., 2007, 2009; Sakata and Vehrencamp 2012). Song stereotypy is determined by calculating the coefficient of variation (CV) = (SD/AVG)*100) using the SDs (standard deviation) of song acoustic features described above and dividing this by the average (AVG) across the same values used to calculate the SD. The CV of song acoustic features reported here is inversely proportional to song acoustic stereotypy.

### Brain collection and verification of implants and castrations

Sixteen days after treatment initiation, birds were deeply anesthetized (4% isoflurane) and weighed, and their brain was extracted and fixed in acrolein after collecting blood from the trunk region into 1.5 ml centrifuge tubes. Blood was centrifuged at 2201 × *g* (8000 rpm for 6 min, and serum was collected and placed at −20°C). Brains were fixed in 5% acrolein PBS under constant agitation for 2 h, then washed four times for 15 min in PBS and cryoprotected in sucrose (30% solution in PBS) overnight until they sank to the bottom of the vial. After cryoprotection, brains were flash frozen in dry ice for 5 min, and then placed in a −70° C freezer. At autopsy, any birds that were incompletely castrated or showed testicular regrowth were removed from the experiment. One bird in the HVC-POM T group and one bird in the PER-T group showed testicular remnants and thus were excluded.

### Brain and serum analyses

Brains were sectioned using a cryostat at 30 μm into four series of coronal sections that were stored in cryoprotectant. These four series were placed in a −20° C freezer. One series was later mounted on gelatin-coated slides and exposed to air for a day. Then, mounted sections were exposed to a standard Nissl staining procedure and coverslipped using Permount (Fisher Scientific). Based on these stained sections, the positions of the implant centers were drawn onto a series of modified atlas plates obtained from the canary atlas made by Stokes et al. (1974) using the revised nomenclature for the songbird brain (Reiner et al., 2004). Another series was used for DCX immunohistochemistry (see below).

Concentrations of serum T were determined using an ELISA (catalog #ADI-900-065, Testosterone ELISA Kit, Enzo Life Sciences) that has been validated in our laboratory for canaries (Madison et al., 2015).This allowed us to determine whether there was any detectable leakage of T from the brain cannula into the peripheral circulation as well as the efficacy of the Silastic implants and castrations.

### Doublecortin immunohistochemistry and quantification

DCX was visualized in one series of brain sections with a DCX immunocytochemistry protocol previously used in canary brains (Boseret et al., 2007; Yamamura et al., 2011). Sections were washed in 0.01 m PBS three times, once in 0.1% sodium borohydride in 0.01 m PBS, and three times in 0.01 m PBS with 1% Triton X (PBST). Endogenous peroxidases were blocked using 0.6% H_2_O_2_ in PBST for 20 min, which was followed by three washes in PBST and additional blocking using 10% normal horse serum (NHS) in PBST for 30 min. Sections were incubated at 4°C in 2% NHS and primary antibody (1:5000 horse-anti goat, doublecortin; catalog #sc-8066, Santa Cruz Biotechnology) in PBST. Sections were washed three times in PBST, incubated in avidin–biotin–horseradish peroxidase complex (1:200, Vectastain ABC Elite Kit) for 1 h, and washed three times in PBST. The peroxidase was then visualized using diamniobenzidine (SIGMAFAST DAB, Sigma-Aldrich) for 5 min, and sections were subsequently washed in 0.01 m PBS and mounted onto gelatin-coated microscope slides. Slides were serially dehydrated in ethanol and placed in xylene for 10 min before being coverslipped using Permount (Fisher Scientific).

We counted separately the two different types of DCX-IR cells—round and fusiform—in HVC. DCX has been shown, based on multiple studies, to be a reliable indicator of new neurons in pallial brain areas (Rao and Shetty, 2004; Boseret et al., 2007; Balthazart et al., 2008), including in HVC, where ∼77% of DCX-positive cells are labeled by bromodeoxyuridine (BrdU) and 73% of BrdU-positive cells are immunoreactive for DCX 10 d after BrdU injections (Balthazart and Ball, 2014a,b). These numbers cannot possibly reach 100%, given the rapid metabolism of BrdU in avian species (Barker et al., 2013) and the presence of non-neuronal elements (glia, endothelial cells) in BrdU-positive cells (Balthazart and Ball, 2014b). Round DCX cells are new neurons that have presumably migrated near their final site and begun differentiating, while the fusiform cells are probably still migrating and have not begun differentiating. The counting method was similar to the method used in previous studies by us and others (Boseret et al., 2007; Balthazart et al., 2008; Yamamura et al., 2011; Alward et al., 2014). Specifically, DCX-IR cells were counted at three different rostrocaudal levels in three separate fields positioned in the center of HVC and in the adjacent nidopallium lateral and ventral to HVC (Balthazart et al., 2008, their Fig. 1). These three rostrocaudal levels of HVC were approximately equally spaced in the nucleus to provide an overall representation of DCX-IR cells in this structure (Balthazart et al., 2008), and they showed significant levels of neuronal incorporation in adulthood (Kirn et al., 1999; Boseret et al., 2007; Balthazart et al., 2008; Yamamura et al., 2011). Immunoreactive cells were manually counted on images digitized through the microscope (20× objective) in a standardized square area (200 × 200 μm) in each brain region of interest with the help of the Cell Counter function of ImageJ software (version 1.40g; Wayne Rasband, National Institutes of Health). DCX-IR cells were classified as round or fusiform by a human observer. The area used for quantification was positioned within the structure of interest in a standard manner using clearly defined brain landmarks, as previously described in detail (Balthazart et al., 2008; Yamamura et al., 2011). All immunoreactive cells that contained a clear unstained nucleus surrounded by stained cytoplasm were counted manually. Cells were counted in both hemispheres to allow for interhemispheric comparisons. One bird from the HVC-POM T group had damage within HVC that precluded an analysis of DCX cells in HVC. We also counted the two cell types in comparable brain regions, lateral and ventral to HVC, to confirm the specificity of changes in HVC. Cell counts in each area (HVC, lateral or ventral to this nucleus) were added across the three rostrocaudal level (i.e., in three 200 × 200 µm fields or 3 × 0.04 = 0.12 mm^2^) and expressed as the number of cells per square millimeter.

### Song control nuclei and POM volume reconstruction

Photomicrographs of Area X, HVC, and RA were taken at 2.5× magnification in the Nissl-stained sections. One bird from the HVC-POM T group had damage to Area X to the point where volume could not be determined, so it was not included in the analysis of Area X volumes. The area of each nucleus was determined in both hemispheres in each section where it appeared using NIH ImageJ. Volumes were determined by multiplying areas by the section thickness, 0.03 mm, summing these values, and then multiplying this value by 4, since only every fourth section was Nissl stained (Bottjer et al., 1986; Bottjer and Dignan, 1988; Alward et al., 2013). We also quantified the volume of POM as it extends from its intermediate position [where the decussatio supraoptica ventralis extends across the basal level of the brain after the septo-mesencephalic tract split disappears] to its caudal level (where the anterior commissure extends across the midline fully). The volume of POM is highly sensitive to the concentrations of T (Riters et al., 2000; Charlier et al., 2008; Alward et al., 2013), with T correlating positively with POM volume. Thus, the volume of POM is a critically sensitive marker of T that is present locally, in the CSF and the general circulation, and was used as an indicator for the efficacy and specificity of our central and peripheral T implants.

### Statistical analyses

ANOVAs were used to assess the effects of treatments on all measures. To avoid arbitrary value assignments to acoustic/stereotypic measures, we compared only the PER-T, HVC-POM T, and POM-T birds in terms of these features collapsed over the final 3 d of treatment. HVC-T and HVC-POM NO T birds were not included in this analysis to avoid using zero values or arbitrary value assignments that are not meaningful. We used mixed-design ANOVAs to assess the effects of treatment and side of T implants. We used planned comparisons (1) to test for the effects of T specifically in HVC on interhemispheric differences in SCS regions and (2) to test for the effects of localized T in the POM on the interhemispheric differences of this nucleus. This approach was used to avoid a type II statistical error, given the strong a priori assumptions about the effects of T in these areas on morphological plasticity (Riters et al., 2000; Brenowitz and Lent, 2002; Charlier et al., 2008; Alward et al., 2013, 2014). The SCS and POM volumes were log transformed to reduce skew. Tukey’s tests were used for *post hoc* pairwise comparisons following significant effects of treatment in the mixed design ANOVAs. Correlational tests were performed using Pearson’s *r* statistic. Effects were considered significant at *p* ≤ 0.05. More details of the statistical analyses are provided in Table 1. Letter superscripts throughout the Results section denote where in Table 1 more information can be found regarding the statistical results.

**Table 1:** Statistical table

Results	Data structure	Test	Power
a; POM volume; treatment × implant side	Normally distributed	Mixed-design ANOVA, Tukey’s	0.9–1.0
b; POM volume; contralateral side	Normally distributed	One-way ANOVA, Tukey’s	1
c; POM volume, ipsilateral side	Normally distributed	One-way ANOVA, Tukey’s	1
d [T]; CP, syrinx mass; treatment	Normally distributed	One-way ANOVA, Tukey’s	0.96–1.0
e; song; stereotypy,variance, energy, song rate	Normally distributed	One-way ANOVA, Tukey’s	0.8–0.99
f; HVC volume; treatment × implant side	Normally distributed	Mixed-design ANOVA, Tukey’s	0.99, 0.75, 0.22*
g; RA volume; treatment × implant side	Normally distributed	Mixed-design ANOVA, Tukey’s	0.72, 0.38, 0.05*
h; area × volume; treatment × implant side	Normally distributed	Mixed-design ANOVA, Tukey’s	0.96, 0.56, 0.19*
i; DCX cells in control regions†; treatment × implant side	Normally distributed	Mixed-design ANOVA, Tukey’s	0.2–0.6, 0.07–0.1, 0.05–0.06*
j; DCX fusiform/round cells; treatment × implant side	Normally distributed	Mixed-design ANOVA, Tukey’s	0.91/0.82, 0.39/0.40, 0.27/0.09*

Data structures for all ANOVAs are listed with the corresponding statistical Power values. Power values were calculated for all aspects of the omnibus ANOVA (i.e., treatment, treatment × implant side, and implant side for mixed-design ANOVAs). The range of power values associated with some tests is listed for certain variables analyzed.

*Power values are listed for treatment, treatment × implant side, and implant side, respectively.

†Statistical information for the four control regions (fusiform and round cells ventral to HVC, and fusiform and round cells lateral to HVC) where DCX cells were counted. For statistical tests on DCX cells, power values are listed as “DCX fusiform/DCX round.”

## Results

### Effects of peripheral versus central T implants

**Figure 1.**
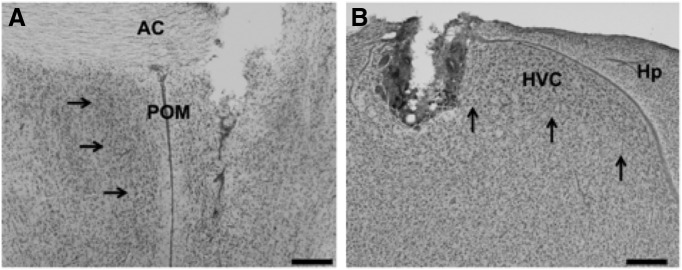
***A***, ***B***, Representative photomicrographs illustrating the typical implant localizations for POM (***A***) and HVC (***B***). Cannulae tips seen here are adjacent to the nucleus of interest to minimize damage to the nucleus. Arrows outline the borders of each nucleus. A Nissl stain was used to visualize the nuclei. Scale bar, 200 μm. Hp, Hippocampus.

**Figure 2.**
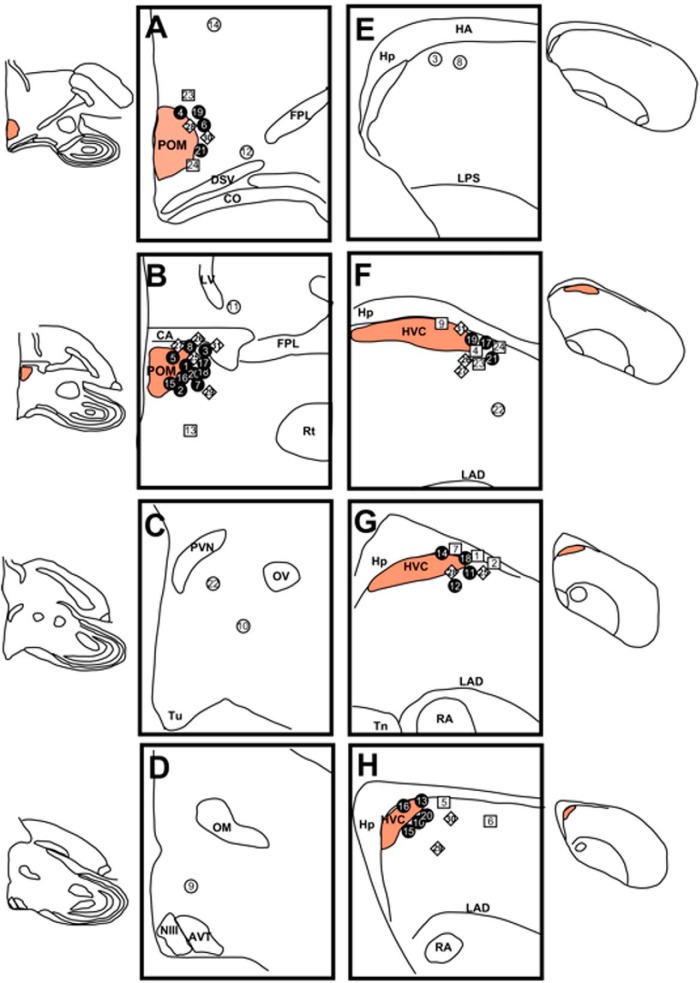
Semi-schematic presentation of the implant locations and their content. ***A–H***, Successive locations in or around POM (***A–D***) or HVC (***E–H***), presented in each case in a rostral-to-caudal order. T-filled implants that contacted their target are represented by black circles, T-filled implants that failed to reach their target are represented by open circles. Empty implants are represented by squares and empty implants in the PER group (birds with a Silastic subcutaneous T-filled implants) are represented by diamonds. Since each bird received two brain implants (T-filled or empty), all symbols are associated with a number that allows reconstructing each pair of implants. In ***A***, ***B***, ***F***, and ***G***, implants near POM or HVC have been slightly spread apart to preserve visibility. AVT, Area ventralis of Tsai; CA, commissura anterior; CO, chiasma opticum; FPL, fasciculus prosencephalis lateralis; HA, hyperpallium apicale; Hp, hippocampus; LAD, lamina arcopalllialis dorsalis; LPS, lamina pallio-subpallialis; LV, lateral ventricle; NIII, nervus oculomotorius; OM, tractus occipitomesencephalicus; OV, nucleus ovoidalis; PVN, nucleus paraventricularis; Rt, nucleus rotundus; Tn, nucleus taeniae of the amygdala; Tu, tuberal hypothalamus.

**Figure 3.**
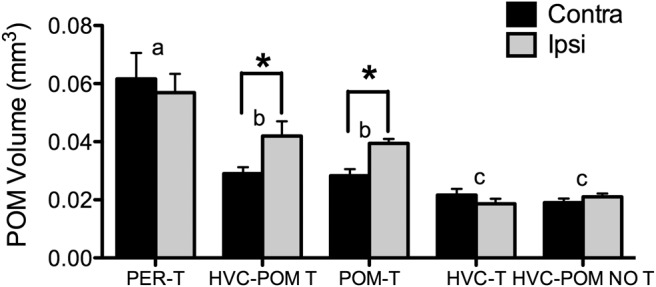
Effects of T implants on POM, HVC, and both on POM volume in the hemisphere implanted with T [ipsilateral (ipsi)] and in the opposite hemisphere [contralateral (contra)]. Bars represent the mean ± SEM. The same letter above individual groups indicates the absence of statistical differences, and different letters above bars indicate a significant difference. The asterisks refer to differences between ipsilateral and contralateral sides. PER-T, *n* = 7; HVC-POM T, *n* = 7; POM-T, *n* = 8; HVC-T, *n* = 5; HVC-POM NO T, *n* = 4. Differences were considered significant at *p* ≤ 0.05.

Representative photomicrographs illustrating the typical implant localizations are presented in Figure 1. Figure 2 illustrates the locations of all of the implants made targeting POM and HVC. The T implants targeting POM were effective, as assessed by changes in the volume of this nucleus (Fig. 3; treatment, ^a^*F*_(4,26)_ =18.49, *p* < 0.05; treatment × implant side, ^a^*F*_(1,4)_ = 9.82, *p* < 0.05; implant side, ^a^*F*_(1,26)_ = 11.61, *p* < 0.05) and only had localized effects (planned comparisons of POM volume, contralateral vs ipsilateral for HVC-POM T and POM-T groups, *t*_6_ = 3.80 and *t*_7_ = 3.68, respectively, *p* < 0.05; *t* ≤ 1.74, *p* ≥ 0.27 for all other paired comparisons; Alward et al., 2013, 2014). *Post hoc* Tukey’s tests revealed that PER-T birds had overall POM volumes that were larger compared to the other groups (*p* < 0.05 for all comparisons); HVC-POM T and POM-T birds were indistinguishable from one another (*p* = 0.99), but both had larger overall POM volumes compared to HVC-T and HVC-POM NO T birds (*p* < 0.05 for both comparisons). However, one-way ANOVAs of data from each side separately showed that, while on the contralateral side PER-T birds had larger POM volumes than all the other groups (^b^*F*_(4,26)_ = 16.46, *p* < 0.05; Tukey’s *post hoc* tests, *p* < 0.05 for all comparisons), which did not differ from one another (*p* ≥ 0.12 for all comparisons), on the ipsilateral side (^c^*F*_(4,26)_ = 18.46, *p* < 0.05) PER-T, HVC-POM T, and POM-T birds did not differ from one another (*p* ≥ 0.12 for all comparisons) yet possessed significantly larger POM volumes than the HVC-T and HVC-POM NO T groups (*p* < 0.05 for all comparisons). These data strongly suggest that implants were effective in inducing unilateral POM growth in POM-T birds similar to that of PER-T birds, and the effects of these implants were localized since they did not affect the contralateral part of the nucleus. T implants in POM or HVC also affected the volumes of song control nuclei in a specific manner, as will be discussed in detail in one of the following sections.

**Figure 4.**
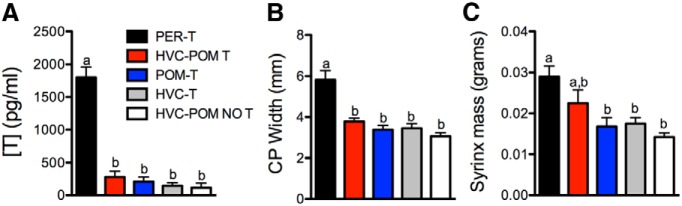
***A–C***, Effects of treatments on serum concentrations of testosterone (***A***) and on two peripheral measures of T action, the cloacal protuberance (CP) width (***B***) and the syrinx mass (***C***). Bars represent the mean ± SEM. The same letter above individual groups indicates the absence of statistical differences, and different letters above bars indicate a significant difference. PER-T, *n* = 7; HVC-POM T, *n* = 7; POM-T, *n* = 8; HVC-T, *n* = 5; HVC-POM NO T, *n* = 4. Differences were considered significant at *p* ≤ 0.05.

There was no detectable leakage of T from the central implants into the general circulation (Fig. 4). Serum T levels differed significantly between groups (^d^*F*_(4,26)_ = 51.70, *p* < 0.05) but *post hoc* comparisons indicated no contribution of central T implants to the systemic concentrations. There were no differences among the HVC-POM T, POM-T, HVC-T, and HVC-POM NO T groups, which all had substantially lower concentrations of circulating T compared with the PER-T group (Fig. 4*A*). PER-T birds had high circulating T concentrations (mean ± SEM, 1801 ± 159 pg/ml) in the range of values seen in sexually active intact birds, and castrates without peripheral T implants had substantially lower circulating levels (pooled mean for all groups, 203 ± 39 pg/ml). These T concentrations are in line with those found in previous studies in canaries (Alvarez-Borda and Nottebohm, 2002; Alward et al., 2013).

Furthermore, the width of the androgen-sensitive cloacal protuberance (Appeltants et al., 2003; Tramontin et al., 2003) was affected by the treatments (Fig. 4*B*; ^d^*F*_(4,26)_ = 14.79, *p* < 0.05), and this width was larger in the PER-T groups than in all other groups (Tukey’s test, *p* < 0.05), which were indistinguishable from one another (all comparisons including those with the HVC-POM NO T group, *p* ≥ 0.50). There was also a main effect of treatment on syrinx mass (Fig. 4*C*; ^d^ *F*_(4,26)_ = 5.86, *p* < 0.05), such that PER-T birds had a significantly larger syrinx mass than POM-T, HVC-T, and HVC-POM NO T birds (*p* < 0.05). Interestingly, HVC-POM T birds were intermediate between PER-T birds and the other groups (*p* = 0.34, and *p* > 0.20 but <0.94, respectively).

### Testosterone in HVC regulates song stereotypy and complexity but not energy

**Figure 5.**
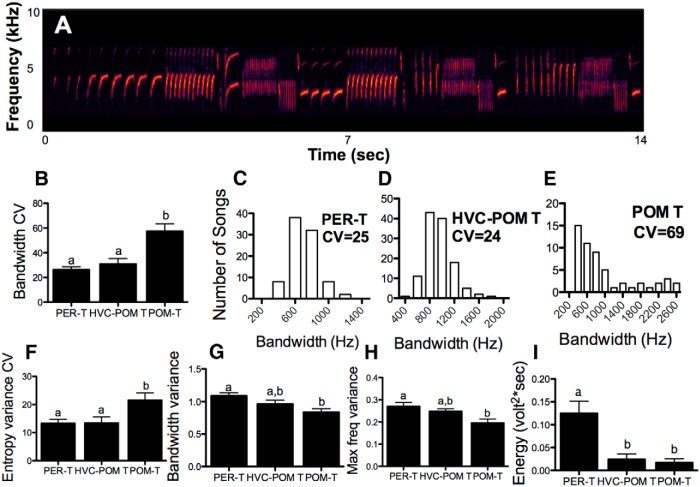
Effects of treatments on various acoustic and stereotypy measures of song. Bars represent the mean ± SEM. The same letter above individual groups indicates the absence of statistical differences, and different letters above bars indicate a significant difference. ***A***, An example of a male canary song. ***B***, ***F***, The effects on measures of song acoustic stereotypy (lower CV means higher stereotypy). ***G***, ***H***, The effects on measures of song complexity (acoustic variance correlates positively with song complexity; see Materials and Methods). ***I***, The effects on song energy (a measure of song amplitude). ***C–E***, Histograms for individual birds from PER-T, HVC-POM T, and POM-T groups, respectively, to demonstrate differences in bandwidth stereotypy (the higher the CV, the lower the stereotypy). PER-T, *n* = 7; HVC-POM T, *n* = 7; POM-T, *n* = 8; HVC-T, *n* = 5; HVC-POM NO T, *n* = 4. Differences were considered significant at *p* ≤ 0.05.

T treatment had significant effects on multiple features of song stereotypy and acoustic structure (Fig. 5; ^e^*F*_(2,19)_ ≥ 4.57, *p* < 0.05 for all). T in HVC increased the stereotypy of songs in birds that also had T in their POM to levels that were indistinguishable from birds with T acting globally (Fig. 5*B–F*). The measures of stereotypy of songs produced by these two groups were about twice as high compared with birds with T solely in their POM. Measures of song complexity, such as bandwidth variance and maximum frequency variance, were also enhanced by T in the HVC of birds with T in their POM, but not to the full level seen in PER-T birds (Fig. 5*G*,*H*). T in POM did not activate these complexity measures of song to the levels of songs produced by PER-T birds. However, neither T in POM nor T in POM plus HVC was able to enhance the energy of songs to the level of PER-T birds; indeed, PER-T birds sang songs that were about three times as loud as in the two other groups (Fig. 5*I*).

Confirming previous findings, T in POM enhanced the song rate to the levels of birds with T acting globally (^e^*F*_(2,28)_ = 5.81, *p* < 0.05; PER-T versus POM-T, *p* = 0.41; PER-T and POM-T vs POM-NO T birds, *p* < 0.05).

### Testosterone and singing activity modulate song control nuclei volumes

**Figure 6.**
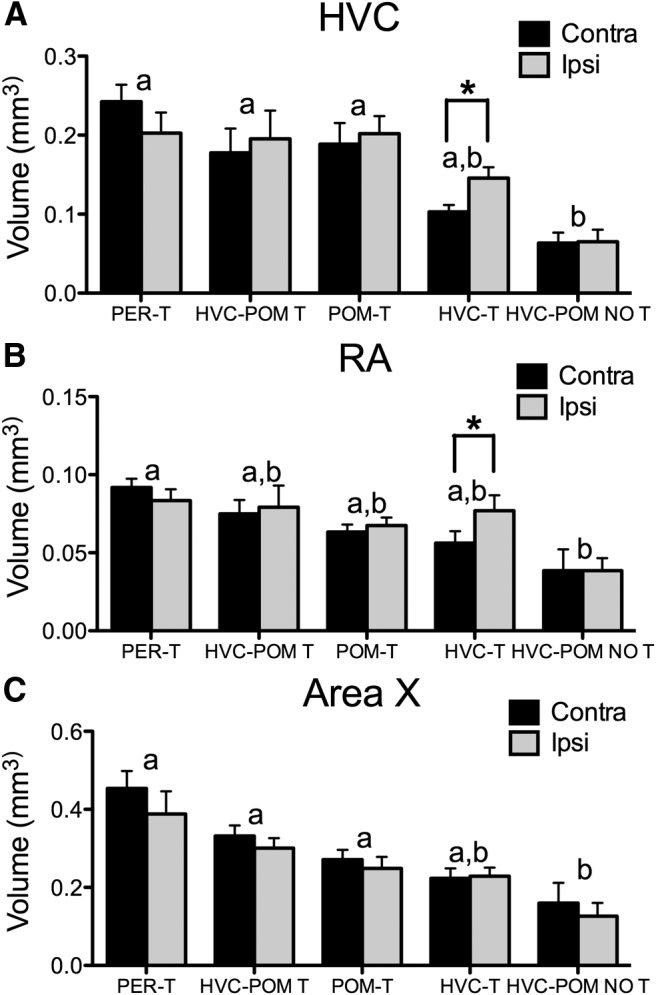
***A–C***, Effects of T implants on the volume of the song control nuclei HVC (***A***), RA (***B***), and Area X (***C***) in the hemisphere implanted with T [ipsilateral (ipsi)] and in the opposite side [contralateral (contra)]. Bars represent the mean ± SEM. The same letter above individual groups indicates the absence of statistical differences, and different letters above bars indicate a significant difference. The asterisks refer to differences between ipsilateral and contralateral sides. PER-T, *n* = 7; HVC-POM T, *n* = 7; POM-T, *n* = 8; HVC-T, *n* = 5; HVC-POM NO T, *n* = 4. Differences were considered significant at *p* ≤ 0.05.

There was a significant effect of treatment on HVC volume (Fig. 6*A*; ^f^*F*_(4,26)_ = 7.14, *p* < 0.05) and an interaction between treatment and implant side (^f^*F*_(1,4)_ = 3.22, *p* < 0.05), but no effect of implant side (^f^*F*_(1,26)_ = 1.53, *p* = 0.23). PER-T, HVC-POM T, and POM-T birds had larger HVC volumes compared with HVC-POM NO T birds (Tukey’s test, *p* < 0.05 for all comparisons) and did not differ from one another (*p* ≥ 0.73 for all comparisons). HVC-T birds did not differ compared with HVC-POM NO T birds but also did not differ compared with all the other groups (*p* = 0.15 and *p* ≥ 0.19, respectively). Planned comparisons (see Materials and Methods) showed in the HVC-T group that the ipsilateral HVC was larger than the contralateral side (*t*_(4)_ = 2.90, *p* < 0.05), but in the HVC-POM T group this hemispheric difference was not observed (*t*_(6)_ = 1.55, *p* = 0.12)

Similar results were obtained for RA. Treatment significantly affected RA volumes (Fig. 6*B*; ^g^*F*_(4,26)_ = 3.03, *p* < 0.05), but an effect of implant side (^g^*F*_(1,26)_ = 0.22, *p* = 0.88; and a significant interaction, ^g^*F*_(1,4)_ = 1.41, *p* = 0.25) was not detected. Tukey’s tests revealed that PER-T, HVC-POM T, POM-T, and HVC-T birds were not different (*p* ≥ 0.62 for all comparisons). PER-T birds had significantly different RA volumes compared with HVC-POM NO T birds (*p* = 0.02); HVC-POM-T and POM-T birds, however, were not different compared with HVC-POM NO T birds (*p* = 0.19 and *p* = 0.18, respectively). HVC-T birds were again not different from any of the other groups (*p* ≥ 0.26 for all comparisons). However, a planned comparison showed that birds implanted with T in their HVC unilaterally had larger RA volumes on the ipsilateral side compared with the contralateral side (*t*_(4)_ =8.91, *p* < 0.05), which was not observed for HVC-POM T birds (*t*_(6)_ = 0.65, *p* = 0.54).

Area X volumes were significantly affected by the treatments (Fig. 6*C*; ^h^*F*_(4,26)_ = 5.80, *p* < 0.05), but there was no interaction between treatment and implant side (^h^*F*_(1,4)_ = 2.18, *p* = 0.10) or an effect of implant side (^h^*F*_(1,26)_ = 1.22, *p* = 0.28. As above, PER-T, HVC-POM T, and POM-T birds had similar Area X volumes (*p* ≥ 0.17 for all comparisons) and had larger Area X volumes compared with HVC-POM NO T birds (*p* < 0.05). HVC-T birds were not different compared with the other groups (PER-T, *p* = 0.09; all other comparisons, *p* ≥ 0.32). Planned paired comparisons within the HVC-T and HVC-POM T groups did not detect interhemispheric differences (*p* ≥ 0.28).

**Figure 7.**
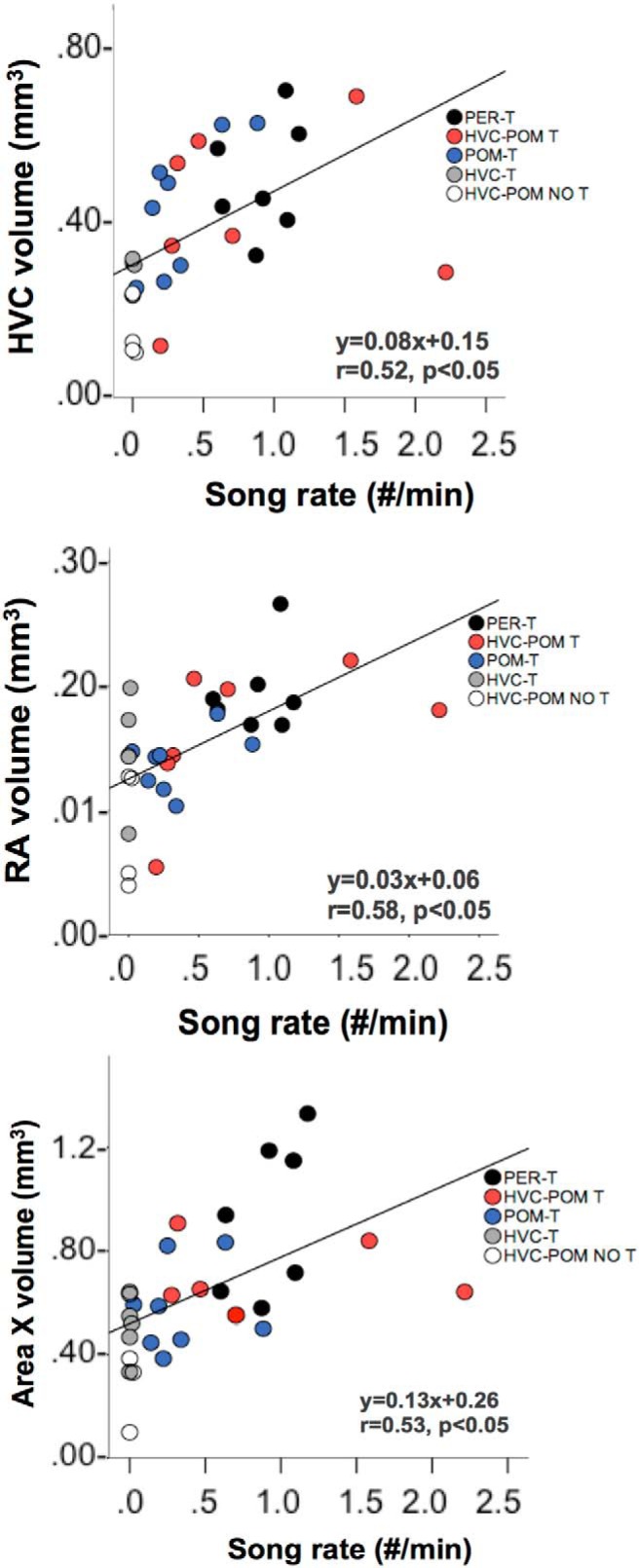
Relationship between song rate (number/minute), and the total volumes of HVC, RA, and Area X. Individual data points from the different experimental groups are coded by different colors. The equation for the regression line, the Pearson’s correlation coefficient *r*, and the associated *p* value are shown in the bottom right portion of each correlation graph. PER-T, *n* = 7; HVC-POM T, *n* = 7; POM-T, *n* = 8; HVC-T, *n* = 5; HVC-POM NO T, *n* = 4.

Last, there was a significant positive correlation between song rate and the volume of HVC, RA, and Area X (Fig. 7).

### Testosterone and singing separately affect the differentiation of new neurons in HVC, but testosterone is required for the recruitment of new neurons

#### Round and fusiform DCX-positive cells in control regions

There was no effect of treatment, implant, or an effect of treatment × implant on fusiform cells (treatment, ^i^*F*_(4,26)_ = 0.97, *p* = 0.44; implant, ^i^*F*_(1,26)_ = 1.64, *p* = 0.21; treatment × implant, ^i^*F*_(1,4)_ = 0.66, *p* = 0.63) or round cells (treatment, ^i^*F*_(4,26)_ = 1.72, *p* = 0.17, implant ^i^*F*_(1,26)_ = 0.43, *p* = 0.52; treatment × implant, ^i^*F*_(1,4)_ = 2.03, *p* = 0.12) in the area ventral to HVC. The same pattern was observed for fusiform cells (treatment, ^i^*F*_(4,26)_ = 0.77, *p* = 0.56; implant, *F*_(1,26)_ = 0.26, *p* = 0.61; treatment × implant, ^i^*F*_(1,4)_ = 0.85, *p* = 0.50) and round cells (treatment, ^i^*F*_(4,26)_ = 0.69, *p* = 0.61; implant, ^i^*F*_(1,26)_ = 0.34, *p* = 0.56; treatment × implant, ^i^*F*_(1,4)_ = 1.88, *p* = 0.14) in the area lateral to HVC.

#### Fusiform DCX-positive cells in HVC

**Figure 8.**
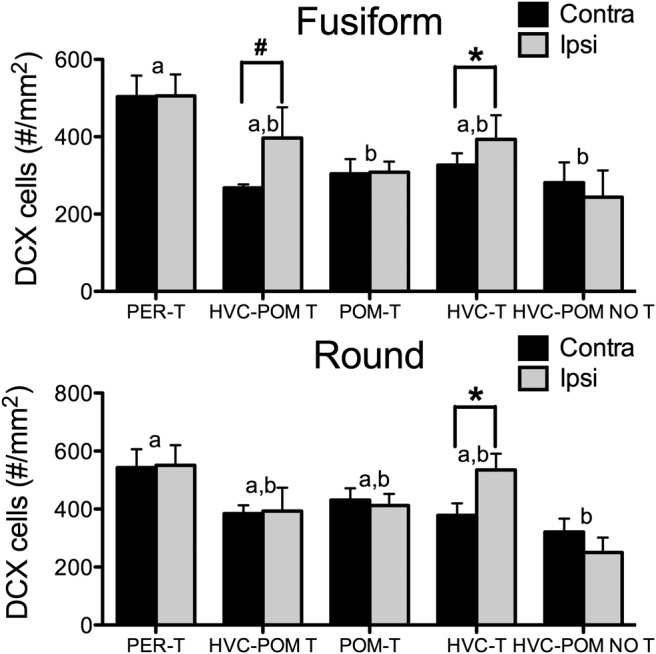
Effects of treatments on the number of DCX-IR cells in HVC. ***A***, ***B***, The numbers of fusiform (***A***) and round (***B***) cells in the hemispheres contralateral (contra) and ipsilateral (ipsi) to the implant site, Bars represent the mean ± SEM. The asterisk refers to a significant difference between ipsilateral and contralateral sides. #A difference between the ipsilateral and contralateral side, but at *p* = 0.08. The same letter above individual groups indicates the absence of statistical differences, and different letters above bars indicate a significant difference. PER-T, *n* = 7; HVC-POM T, *n* = 6 (one bird had damage within HVC that precluded DCX quantification; see Materials and Methods); POM-T, *n* = 8; HVC-T, *n* = 5; HVC-POM NO T, *n* = 4. Differences were considered significant at *p* ≤ 0.05.

The number of fusiform DCX cells in HVC was significantly affected by the treatments (Fig. 8*A*; ^j^*F*_(4,25)_ = 4.76, *p* < 0.05). Specifically, PER-T birds had more fusiform cells compared with POM-T and HVC-POM NO T birds (Tukey’s test, *p* < 0.05 for both). HVC-POM T and HVC-T birds were not different compared with each other or with the other groups (*p* ≥ 0.22). There was no statistical effect detected for implant side (^j^*F*_(1,25)_ = 1.39, *p* = 0.24), and no interaction between implant side and treatment (^j^*F*_(1,4)_ = 1.57, *p* = 0.21). However, a planned paired comparison testing the specific effects of local T in HVC (see Materials and Methods) showed that HVC-T birds possessed significantly more fusiform cells in the ipsilateral than the contralateral HVC (*t*_(4)_ = 3.60, *p* < 0.05); HVC-POM T birds also tended to have more fusiform cells in the ipsilateral HVC relative to the contralateral HVC (*t*_(5)_ = 2.14, *p* = 0.08).

#### Round DCX-positive cells in HVC

Testosterone treatment had a significant effect on DCX round cells in HVC (Fig. 8*B*; ^j^*F*_(4,25)_ = 3.81, *p* < 0.05). PER-T birds had more round cells in HVC than HVC-POM NO T birds (Tukey’s test, *p* < 0.05). No other differences between groups were detected (*p* ≥ 0.12 for all comparisons). There was no effect of implant side (^j^*F*_(1,25)_ = 0.35, *p* = 0.56). There was also no statistical effect detected for the interaction between implant side and treatment (^j^*F*_(1,4)_ = 1.52, *p* = 0.20). In the HVC-T group, a planned within-subjects comparison revealed an interhemispheric difference such that the HVC ipsilateral to the T implant had more round cells than the contralateral HVC (*t*_(4)_ = 7.07, *p* < 0.05). This difference was not observed for the HVC-POM T group (*t*_(5)_ = 0.10, *p* = 0.92).

#### Correlation with singing activity

**Figure 9.**
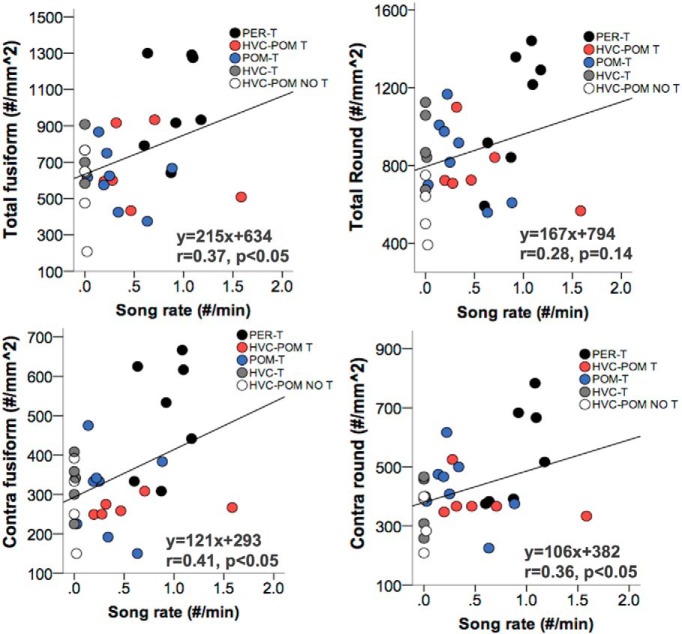
***A***, ***B***, Relationship between song rate (number/minute) and the numbers of DCX-IR cells in HVC calculated separately for the two types of DCX-IR cells, fusiform (***A***) and round (***B***), across hemispheres (***A***, ***B***) and on the contralateral (Contra; ***C***, ***D***) side. Individual data points from the different experimental groups are coded by different colors. Correlations were calculated using Pearson’s *r*. The equation for the regression line, the Pearson’s correlation coefficient *r*, and the associated *p* value are shown in the bottom right portion of each graph. PER-T, *n* = 7; HVC-POM T, *n* = 6 (one bird had damage within HVC that precluded DCX quantification; see Materials and Methods); POM-T, *n* = 8; HVC-T, *n* = 5; HVC-POM NO T, *n* = 4.

There was a significant positive correlation between song rate and the total number of fusiform cells (Fig. 9). There was a positive correlation between song rate and the total number of round cells in HVC, but this was not significant (*p* = 0.14). The correlation between song rate and DCX cells in the contralateral HVC was also assessed to remove the effects of local T on the ipsilateral side (in HVC-POM T and HVC-T birds) that could confound the relationship between song rate and total DCX cells. This correlation revealed a significant positive relationship between song rate and the number of fusiform and round cells in the contralateral HVC.

## Discussion

Steroid hormones have prominent effects on behaviors and their neural substrate. Here we elucidate how different features of a single behavior can be regulated by separate actions of the same hormone in distinct regions of the brain. Moreover, we show that hormones can regulate neuroplasticity by acting directly at target sites (e.g., the effect of HVC-T implants on HVC plasticity) or acting transsynaptically (e.g., the effects of HVC-T implants on RA plasticity), as well as via activity-driven mechanisms (e.g., the effects of POM-T implants on HVC plasticity).

### Testosterone must act at multiple levels to regulate the different features of birdsong

We first replicated previous work showing that T acting in POM is sufficient to increase song rate but not other attributes of song behavior, such as its stereotypy and energy (Alward et al., 2013). We then hypothesized that T needs to act in HVC to enhance song stereotypy to levels observed in PER-T birds (Meitzen et al., 2007). To test this notion, we implanted T in HVC of birds that had T also in POM. We found that T in HVC is sufficient to increase song stereotypy that was absent in birds with T in their POM only. We also showed that T in HVC plays a role in regulating the within-song complexity that was increased to levels intermediate between those seen in birds treated with T systemically and birds only exposed to T in POM.

HVC expresses both androgen and estrogen receptors (the latter being observed only in some species, including canaries; Bernard et al., 1999; Metzdorf et al., 1999; Ball et al., 2002) . Based on studies in zebra finches, the majority of these androgen-positive neurons in HVC project to either RA or Area X (Sohrabji et al., 1989). The changes in song quality that we observed could be driven by the actions of T on androgen receptor-positive neurons that project to either RA or Area X. Most likely, the actions of T in HVC in the control of song stereotypy and complexity occur as a product of the transsynaptic regulation of multiple effector sites, including RA and Area X. For instance, it is reasonable to assume that the actions of T (and its androgenic metabolite, 5-alpha dihydrotestosterone) on androgen receptor-positive HVC neurons projecting to Area X drove the enhancement in song stereotypy. Area X is part of the subcircuit within the SCS, ultimately involving LMAN projections to RA, that controls the variation between song renditions (Kao et al., 2005; Brainard and Doupe, 2013; Kojima et al., 2013). Within-song complexity also is presumably regulated by the same projections (Rouse and Ball, 2015). HVC neurons, and in particular neurons located in the lateral portion of this nucleus, have been shown to encode for individual syllables (Basista et al., 2014). Since HVC-T implants led to an increase in within-song complexity (albeit subtle) and our T implants near HVC were adjacent to the lateral portion of this nucleus, it is plausible that the enhancement of within-song complexity observed here was due to the actions of T on HVC neurons that encode for different syllables.

Still, the T in HVC was not sufficient to increase within-song complexity to the level observed in birds exposed to T acting globally. One possible explanation for this observation is that our T implants adjacent to the lateral portion of HVC did not diffuse broadly enough to affect all HVC neurons that control within-song complexity. However, HVC-T implants fully enhanced song stereotypy to levels present in PER-T birds, suggesting that our T implants affected a sufficient part of HVC to be behaviorally effective. An intriguing hypothesis is that T must act at multiple levels of the SCS, including, for example, RA, LMAN, and/or the syrinx (i.e., regions that express androgen receptors and are involved in the control of within-song variability; Yu and Margoliash, 1996; Fee et al., 1998; Elemans et al., 2009; Woolley et al., 2014; Rouse and Ball, 2015) to enhance complexity. Especially important for the control of within-song variability may be the connection from RA to tracheosyringeal hypoglossal nucleus (nXIIts) to the syrinx, given that the syrinx is myotopically innervated by the RA–nXIIts projection (Vicario and Nottebohm, 1988; Vicario, 1991). Indeed, the syrinx must undergo complex morphological changes to produce complex birdsong, a process that is tightly linked to the innervation by RA via nXIIts and presumably under the control of T (Luine et al., 1980; Vicario, 1991; Fee et al., 1998; Veney and Wade, 2004; Elemans et al., 2009). Hence, T in HVC may be able to enhance within-song complexity—most likely via transsynaptic actions on RA (Meitzen et al., 2007)—but the full enhancement of song complexity requires the actions of T at other levels of the SCS.

Whereas T action in HVC enhanced song stereotypy and, to some extent, within-song complexity, this treatment had no effect on song loudness, and both HVC-POM T and POM-T birds sang with about three times less energy than PER-T birds. Research on the regulation of the amplitude of vocalizations indicates that respiratory activity plays a critical role (Wild et al., 1998; Franz and Goller, 2002; Plummer and Goller, 2008). Nuclei involved in coordinating respiratory activity and singing, such as the nucleus retroambigualis and nuclei of the ventral respiratory group in the hindbrain, express androgen receptors in zebra finches (Gahr and Wild, 1997), and T action at this level of the SCS is probably needed to enhance song amplitude. The syrinx, another androgen-dependent structure (Lieberburg and Nottebohm, 1979; Luine et al., 1980; Bleisch et al., 1984; Veney and Wade, 2004) could also play a role in regulating song energy (Elemans et al., 2009). Thus, it appears likely that the amplitude of songs is controlled by androgens acting outside HVC or POM at yet another site of action located in the hindbrain or periphery.

### Testosterone and singing activity influence neuroplasticity in the song control system

Previous studies have shown that steroid hormones have robust effects on the morphology and physiology of the SCS (for review, see Schlinger and Brenowitz 2009). For example, Brenowitz and Lent (2002) found that local implants of T increase the volume of the adjacent HVC and enhance the size of the downstream nuclei RA and Area X on the ipsilateral side. Other studies have shown that the act of singing itself can promote the release of brain-derived neurotrophic factor (BDNF) in HVC that results in increases in HVC volume and/or increases in the incorporation of new neurons into HVC (Li et al., 2000; Alvarez-Borda and Nottebohm, 2002; Alward et al., 2013). It is important to note that BDNF is a major target gene for T action in HVC that results in plasticity changes (Rasika et al., 1999). Thus, activity-dependent effects and direct T effects on HVC plasticity have at least one key plasticity gene in common as a target. T by itself can be sufficient to induce the full range of seasonal changes in song system structures (Brenowitz and Larson 2015), but other factors such as singing activity can also upregulate BDNF concentrations. Here we provide evidence in support of both of these phenomena. T implanted in HVC enhanced the volume of the ipsilateral HVC and RA in the absence of singing activity. In addition, T implanted in POM activated singing, and the expression of the behavior itself presumably was able to enhance the volumes of SCS nuclei, as previously shown (Alward et al., 2013).

The direct action of T in HVC also increased the number of fusiform and round DCX-positive cells in HVC, suggesting that the local action of T promotes the attraction and incorporation of new neurons into this nucleus. There was also a similar trend for round, but not for fusiform, DCX-positive HVC cells among birds in the POM-T group, suggesting that another factor, perhaps singing activity, could be able to modulate the incorporation/differentiation, but possibly not the recruitment/migration, of new neurons into the nucleus. This suggestion is supported by the fact that the number of round cells in HVC among birds in the POM-T group was intermediate between the PER-T and HVC-POM NO T groups. Previous work had suggested that gonadal steroids and singing activity can independently and additively modulate the incorporation of new neurons into HVC (Alvarez-Borda and Nottebohm, 2002). The present results support these ideas by independent methods and indicate that T acting directly in HVC modulates new neuron recruitment and incorporation, while singing activity also can enhance the incorporation of these neurons into HVC. Importantly, we replicated, based on the quantification of DCX-positive neurons, the work by Li et al. (2000), based on incorporation of the thymidine analog BrdU, showing that singing activity positively correlates with the incorporation of new neurons into HVC.

There was a slight increase in the syrinx mass of HVC-POM T birds. We do not think this increase was due to the leakage of T from our central implants, given the absence of effects of brain T implants on (contralateral) POM or HVC volumes, on cloacal protuberance width, and on circulating T concentrations. Instead, this increase in syrinx mass may be due to transsynaptic effects of T acting in HVC combined with the effects of singing activity. This effect on the syrinx mass was indeed not observed in birds with T solely in their HVC or with T solely in their POM, suggesting that this effect cannot be driven solely by local T action in HVC or by singing activity alone. The increase in syrinx mass may result from T action in HVC, but the actual use of the syrinx (i.e., singing) would be a permissive factor for this increase to occur. Transsynaptic effects of T in HVC on RA were observed here and by Brenowitz and Lent (2002); it is possible that these effects proceed downstream to the syrinx if the bird is singing. Rand and Breedlove (1995) have shown similar androgen-dependent transsynaptic relationships between the spinal nucleus of the bulbocavernosous and its target muscles in the penis in rats. These observations are in line with ideas set forward by others that steroid hormones act directly at target sites as well as transsynaptically to exert their effects on neuroplasticity (Beyer and Feder, 1987; Brenowitz, 2015).

### Steroid hormones act in a pleiotropic manner to regulate sociosexual behaviors

Social behaviors are made up of suites of traits and neural mechanisms that need to be intricately coordinated for a behavioral trait to be adaptive (Pfaff et al., 2008). For instance, depending on factors such as seasonal or breeding state, an animal must emphasize certain features of behavior to differing degrees to ensure optimal performance. The nervous system has distributed functional sites throughout the brain as well as the periphery to enhance the computational control over these social behaviors, and in many species hormone receptors are distributed across these functionally discrete areas. Given these constraints, it has been posited that steroid hormones must act nonredundantly (i.e., at different sites with distinct functions) to regulate behavior and physiology (Arnold, 1981; Pfaff et al., 2008) to increase computational control over adaptive behaviors.

This conceptual framework was proposed by McEwen et al. (1979), Pfaff et al. (1994, 2008), and Lee and Pfaff (2008), who implemented a research program to elucidate the neuroendocrine bases of the lordosis posture in female rats. They used a “reverse-engineering” approach in the analysis of these processes, and, based on experimental results, they proposed the existence of different neural modules that constitute the neural circuit that regulates lordosis. They also indicated that some of these modules are estrogen sensitive. Engaging in the lordosis reflex in response to male somatosensory input is one clear behavioral indicator of female sexual receptivity, which is one dimension of a complex of female sexual behaviors that are exhibited by females as a function of many endogenous and exogenous factors (Beach, 1976). Telencephalic inputs to the well defined lordosis circuit are certainly important in the modulation of female sexual behavior, but these do not appear to be modulated by estrogens. One of the surprising aspects of the distribution of AR in the songbird brain is that there are prominent telencephalic nuclei expressing high densities of AR, which is not the case for non-songbird avian species or mammalian species (Arnold et al., 1976; Balthazart et al., 1992). The analysis of singing behavior and its neuroendocrine control thus provides an opportunity to uncover broader principles of neural integration that concern at the same time more complex behaviors and a larger number of brain structures, including steroid-sensitive telencephalic areas.

However, some modules proposed by Pfaff et al. (1994, 2008) are similar to what we observe in songbirds. For instance, the lower brainstem module and the spinal cord module, which are proposed to handle “local business” by Pfaff et al. (1994, 2008), are analogous to projections from nXIIts to the syrinx to regulate different syringeal muscles. One major difference between the lordosis and the song control pathways is that the lordosis circuit is regulated only by hormones in the hypothalamic and the midbrain module, while in the song system there are at least seven major sites of potential androgen regulation, namely, the song control nuclei HVC, RA, LMAN, the nucleus retroambigualis, and the nuclei of the ventral respiratory group in the hindbrain, POM, and syrinx (Bernard et al., 1999; Metzdorf et al., 1999). The next generation of studies will require an investigation of the types of genes whose expression is regulated by sex steroid hormones in different components of neural circuits that control social behavior and assess whether there are generalities at this level of analysis. The song control system may provide a useful model system for such studies.

The results of the current study provide a significant contribution to our understanding of the general phenomenon of the pleiotropic function of steroid hormones but add to these ideas in important ways. As with the observations made by Pfaff et al. (2008) in terms of the estrogenic control of lordosis, it appears that T needs to act at multiple levels in the brain and periphery to regulate all components of a single behavior, song. However, our study reveals dissociable roles of T in telencephalic nuclei (i.e., HVC), diencephalic nuclei (i.e., the POM), and other still unidentified sites, and suggests that the adaptive nature of complex sociosexual behaviors relies on the integration across hypothalamic and telencephalic nuclei. Last, in canaries, HVC expresses estrogen receptors, and the enzyme aromatase, which converts T to estrogens, is densely expressed in the neighboring nidopallium (Metzdorf et al., 1999). This raises the possibility that some of the effects of T on song stereotypy and complexity may be driven by the actions of estrogenic metabolites of T in addition to or instead of androgenic metabolites. There is, indeed, evidence that canary song can be influenced by the actions of estrogenic metabolites (Fusani and Gahr, 2006).

### Conclusion

For decades it has been known that the different components of sociosexual behaviors are modulated by steroid hormone actions. However, it is less clear how distinct traits of social behaviors, including behavioral and physiological changes, are coordinated by differential steroid hormone action. Using the neuroendocrine system underlying male canary birdsong as a model, we have shown that T acts in a pleiotropic manner to regulate birdsong and its underlying neural substrate. These results highlight the complex interrelationships between steroid hormone action, behavior, and neuroplasticity.

## Synthesis

The decision was a result of the Reviewing Editor Jeffrey Blaustein and the peer reviewers coming together and discussing their recommendations until a consensus was reached. A fact-based synthesis statement explaining their decision and outlining what is needed to prepare a revision is listed below. The following reviewers agreed to reveal their identity: Elizabeth Adkins-Regan

The reviewers are in agreement that there a good deal of merit to these studies. There are several relatively minor issues delineated below. However, the one major issue regards how song stereotype and similar values were calculated. The reviewers found it difficult to compare this work to previous studies in this system. Are the stereotypy measures reflecting mainly changes between songs in which syllables are sung, changes in how they are sung, or changes in how many syllables of each type are sung (how long the strings are)? Considering that differentiating between stereotype, rate and amplitude is central to the interpretation, this was considered quite important. This will require adding more details to the methods section.

Other comments:

Reviewer 1:

Line 145: change to read "birds with empty cannulas targeting the HVC and POM and birds with T-filled cannulas that missed"

Line 199: multiplied?

Line 210: there is a parenthesis missing

Lines 218 and 293: analyses

Lines 348-351: what immediately follows the heading doesn't seem to fit the heading

Lines 397-400: there is something missing in this sentence. Are these supposed to be the results for the area lateral to HVC? What are they the results for?

Figure 6 legend: it would be good to repeat the definition of song rate again here. The methods section says it's #/min.

Figure 8B: why does this one give song rate as #/hr on the X-axis?

Reviewer 2:

Methods

1. Lines 205-206. Are the inverse values of CV defined as "stereotypy?" More description of the methods and definitions of the terminology used in this section are necessary for reader comprehension. Please see comments regarding Figure 4 below.

2. Statistics. It appears that the eNeuro required statistics information/table is missing?

Results

3. Lines 363-388. Figure references appear to be missing from this section.

Discussion

4. Role of testosterone metabolites. Testosterone can be metabolized into both other androgens and estrogens. The third paragraph of the discussion partially addresses this, but seems to under-emphasize the role of estradiol. Estradiol is especially relevant given the presence of estrogen receptors in HVC, reports of aromatase expression in HVC, and the links between estradiol and BDNF/growth factor reported in other systems.

Figures - general comments

5. Figure Legends. In some figure legends no descriptions of the subpanels are included. This contributes to reader confusion, especially for Figure 4.

6. Authors: Please note that this is something to consider, but will not be required....Lack of raw data. Figure 1 is excellent for showing the general location of the cannulas, but no other raw images are shown in the paper. The impact of the manuscript would be increased if, for example, songs of differing stereotypy, and/or DCX and song region volume nissl stain immunocytochemistry were shown.

7. Experimental N. If experimental "n" was indicated in the figures it would increase clarity for the reader.

8. Diagrams/Schematics. It is sometimes difficult to navigate the hormone manipulations for each specific experiment. Providing a schematic showing each treatment may help improve clarity. For example, a schematic of a cannula with hormone near HVC could be shown for the HVC-T group, etc.

Figure 4

9. The acronyms are difficult to navigate on this figure (especially 4B). If there's space, writing out the entire word would be helpful to the reader.

10. It is difficult to compare the results in the current paper to previous work given that the "stereotypy" designation is used rather than CV. For example, Bandwidth CV reported in Figure 2F of Alward et al (2013) is about 25 in the PER-T group, but bandwidth stereotypy is about 400 in the present manuscript. If stereotypy is the inverse of CV, then 1/400 works out to be 0.0025, which seems to be orders of magnitude different! So this is very confusing. Are the values really this different or is there a mathematical error in either my or the author's calculations? Ultimately, whether my back of the envelope calculation is correct or not, additional information about the methods generating the data presented in this figure need to be added to the manuscript to avoid this confusion. The authors may also consider using parameters previously reported in the literature in order to better compare data across studies.

11. Similarly, while trying to understand how the stereotypy measurements were generated, I noticed that the values for Energy seemed to be different than those reported in an earlier study in this system (Alward et al., 2013). The mean energy for the PER-T group was about 0.05 in Figure 2 of Alward et al (2013), but is about 0.14 in the present work. Is there any particular reason why this value is so different (i.e., differences in recording technique, etc)?
